# Supplementary material for: Disposable Electrochemical Biosensor Based on the Inhibition of Alkaline Phosphatase Encapsulated in Acrylamide Hydrogels
Source: Biosensors (Basel). 2022 Aug 29;12(9):698. doi: 10.3390/bios12090698 (PMC9496573; doi:10.3390/bios12090698)
Supplement: Supplementary file 1 [file biosensors-12-00698-s001.zip › biosensors-1820380-supplementary.pdf]

Supporting information for

Disposable Electrochemical Biosensor Based on the Inhibition of Alkaline Phosphatase Encapsulated in Acrylamide Hydrogels

Yolanda Alacid [1], Andrés F. Quintero Jaime [2], María José Martínez-Tomé [1], C. Reyes Mateo [1] and Francisco Montilla [2,*]

[1] Instituto de Investigación, Desarrollo e Innovación en Biotecnología Sanitaria de Elche (IDiBE), Universidad Miguel Hernández, Avenida de la Universidad, s/n. 03202 Elche, Alicante, Spain
[2] Departamento de Química Física and Instituto Universitario de Materiales de Alicante (IUMA), Universidad de Alicante, Carretera San Vicente s/n, 03690 Alicante, Spain
* Correspondence: francisco.montilla@ua.es

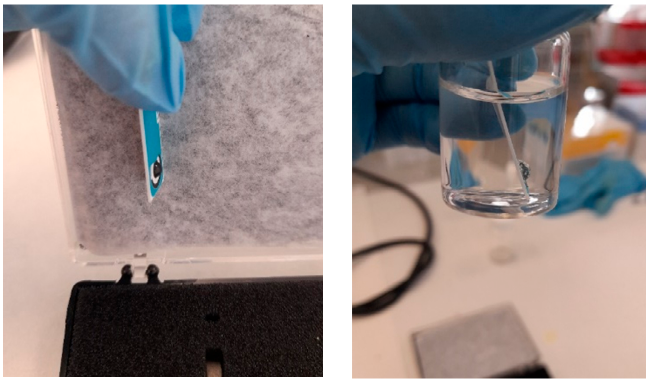

Figure S1. Photographic images of the AETAC hydrogels adhered to the working electrode of the SPE.

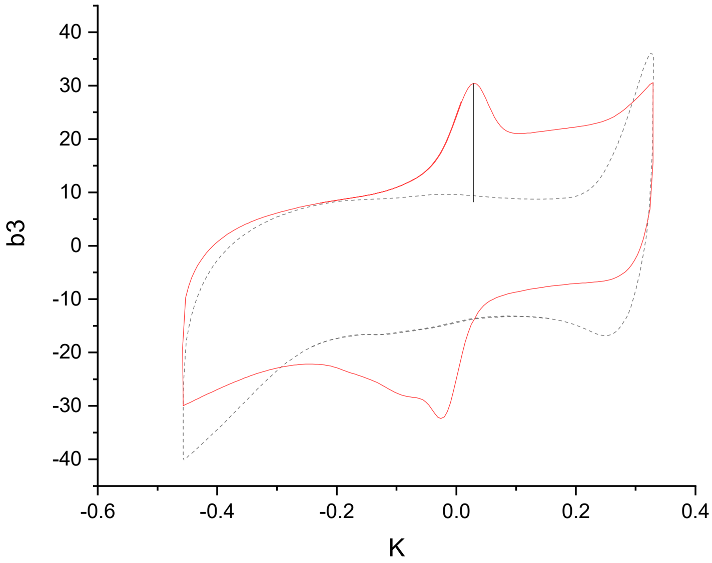

Figure S2. Cyclic voltammograms recorded with *ALP@AETAC*-modified electrode in a solution of 0.5 mM HQ2P in trizma buffer solution containing sodium chloride (50 mM), sodium sulphate (50 mM) and sodium nitrate (50 mM). Dashed line: Initial voltammogram in the presence of the substrate. Solid line: After 20 min of the addition of the substrate.
